# Supplementary material for: Are carbon emissions trading and green financial instruments synergistic? -Comprehensive quantitative research based on content analysis
Source: PLoS One. 2024 Mar 7;19(3):e0298601. doi: 10.1371/journal.pone.0298601 (PMC10919601; doi:10.1371/journal.pone.0298601)
Supplement: S1 Data — (ZIP) [file pone.0298601.s001.zip › Data/地级市Z_F工作报告.zip!/╣π╢1⁄2/╟σ╘╢╩╨/└┤╫╘╣1⁄2╓┌║┼í╛┬φ┐╦╩2╛▌═°í┐_2009.docx]

各位代表：

我代表市人民政府，向大会作政府工作报告，请予审议，并请政协各位委员和其他列席人员提出意见。

2008年工作回顾

2008年是我市发展进程中极不平凡的一年。全市各族人民深入贯彻落实科学发展观，大力推进工业园区化、农业产业化、城镇特色化和管理人性化，成功战胜低温雨雪冰冻灾害，积极应对国际金融危机的不利影响，奋力拼搏，扎实工作，基本完成了市五届人大三次会议确定的各项预期目标。

——经济实现平稳较快发展。全市生产总值、财政一般预算收入、全社会固定资产投资、社会消费品零售总额四项指标增幅居全省首位，实现经济增长全省“五连冠”目标。全年完成生产总值746.6亿元，增长18.4%；人均生产总值2.02万元，增长16.5%。规模工业总产值1620.4亿元，增长42.9%；规模以上工业增加值389.7亿元，增长30.8%。完成全社会固定资产投资703.5亿元，增长44.4%。来源于清远的财政总收入89.1亿元，增长38.3%；其中，财政一般预算收入37.4亿元，增长34%；人均财政一般预算收入1011元，增长31.9%。外贸出口13.8亿美元，增长16.1%。以旅游业为龙头的第三产业加快发展，全年接待游客1488万人次，增长12.5%；旅游收入56.3亿元，增长17.5%；城乡市场兴旺，实现社会消费品零售总额250.6亿元，增长27.3%。物价和金融运行保持稳定，居民消费品价格水平上涨4.5%。年末金融机构人民币各项存款余额645.1亿元，比年初增长15.2%；贷款余额273.9亿元，增长21%。民营经济蓬勃发展，新增个体工商户1.8万户，新增注册资金5.1亿元；新登记私营企业1170家，新增注册资金16亿元。民营经济共完成投资368.6亿元，增长64%，占全社会固定资产投资总额的52.4%；规模以上民营工业企业达399家，完成增加值165.8亿元，增长31.5%，占规模工业的42.5%。

——农业农村经济稳步发展。成功抗击了雨雪冰冻和洪涝灾害，农林牧渔业总产值154.1亿元，增长6%。549户雪灾全倒户、1100户因洪灾需恢复重建的农户全部按期迁入新居。80个新农村示范村建设加快推进，农村饮水安全工程建设全面铺开，共解决饮水不安全问题人口10.2万人；村村通公路建设完成1400公里；新增农村沼气池3200个；2006年和2007年省人大议案农田水利基本建设项目全面完成，2007年基本农田整治项目顺利推进。大力扶持和培育农业产业化经营组织，新增国家扶贫农业龙头企业1家，省农业龙头企业4家。“五大特色农业产业带”和“四大特色农业基地”规模进一步扩大。创品牌成效明显，清新冰糖桔、英德麻竹笋、连南无核柠檬等3个产品获国家地理标志产品保护；新增无公害农产品产地认定企业7家，产品6个；新增绿色食品A级认证产品5个；成功申报4个国家级、8个省级农业标准化示范区。扶贫开发扎实推进，将帮扶责任落实到了个人。建立了贫困户发展生产资金扶持机制，并在全市22个行政村推行。革命老区建设、产业化扶贫、智力扶贫深入实施并不断取得成效。

——城乡建设实现新改观。基础设施明显改善，清远大桥抢修加固工程顺利完成，城西大道一期工程、清连高速公路主线路面已完工并投入试运行；26项列入省城乡水利防灾减灾工程已全部动工；239宗列入省农村机电排灌议案工程已完成98宗；500千伏贤令山、220千伏燕河等一批输变电工程已完工。城市建设与管理水平有效提高，市区小区道路改造、清远职业技术学院直通路延伸段及连接线等一批市政道路已完工；一批民生关注的公厕、青山垃圾填埋场二期工程、飞来湖防灾减灾综合工程等前期工作准备就绪；市区廉租房、101工程等市政工程已动工建设；市区美化、绿化、亮化水平进一步提升，绿化覆盖率达36.95%，绿地率达33.78%；启动了“市肺”工程，共完成更新造林533公顷。城市规划和管理工作进一步加强，土地利用总体规划修编工作全面启动，“一江两岸”城市设计等专项规划编制工作已完成；“城乡清洁工程”深入开展，城市“脏、乱、差”现象得到有效遏制。环境保护取得重大突破，共关闭淘汰小火电、立窑水泥等落后企业130家，完成省政府下达的污染减排年度任务；预计全市单位生产总值能耗下降5.7%；去年9月，省环保局正式解除对清城区有关建设项目的区域限批；加快环保基础设施建设，全市污水处理能力已达12万吨／日；建成了清远市污染源在线监控平台，全市10家国控重点污染源全部实现了在线监控联网，主要江河水质总体保持良好。加大违法违规用地专项整治，积极推进土地节约集约使用，用地需求矛盾逐步缓解。

——“双转移”工作强势推进。园区建设取得突破性进展，佛山（清远）产业转移工业园成功创建为省示范性产业转移园，佛山禅城（清新）产业转移工业园被批准为省产业转移园。广东华侨经济产业园、广东民族工业园等大型产业转移园及各县（市、区）重点园区建设全面启动并加快推进。招商引资成果丰硕，全年引进新项目463个，合同投资金额755.5亿元，增长38.9%，其中投资额超亿元项目114个；累计实际投入资金232.2亿元。全市新批外资项目83个，实际利用外资5.1亿美元，增长15.4%。在全省率先举办产业和劳动力“双转移”培训试点班，与佛山市建立了劳动力对口转移就业合作机制，共培训农村劳动力3.6万人，转移农村劳动力12.7万人次。

——社会各项事业取得新进展。全面实施城乡免费义务教育，54.7万名学生享受免费义务教育；农村学校危房改造工程全面竣工，清城区和佛冈县率先提前两年完成义务教育规范化学校建设省定指标任务；新创建省教育强镇7个，排全省第9位、非珠三角地区第1位；增加中小学优质学位2.9万个。高中阶段毛入学率达63.9%，增幅居全省首位；职业教育水平进一步提高，全市中职在校生达7.1万人。科技、文化等社会事业加快发展，建成了首家省级工程技术研究开发中心，获省认定高新技术企业11家，清城区石角镇、英德市西牛镇被认定为省技术创新专业镇；知识产权和信息化工作进一步加强，政府门户网站建设上新台阶，电子监察系统应用已普及到各县（市、区）及部分乡镇。群众性文体活动深入开展；农村中央广播电视节目无线覆盖工程全面完成，新闻出版事业加快发展。公共卫生体系进一步完善，市人民医院新院区主体工程基本完成，市疾控中心新卫生检验中心已投入使用；乡镇卫生院建设得到加强，村卫生站的配套建设基本完成。积极做好人口和计划生育工作，全市人口自然增长率为6.06‰；创“两无”（镇街无政策外多孩出生、村居无政策外出生）活动取得新进步。深入开展拥军优属，全面落实优抚安置政策，再度荣获“省双拥模范城”称号。民主法制和精神文明建设进一步加强，完成了市直单位行政审批事项的清理和行政执法职权的核准、界定、公告工作，顺利通过了省政府依法行政工作专项检查；积极推行网上“阳光”审批，市直单位行政审批权逐步纳入市电子监察系统监管范围，审批事项提前办结率达95.4%。圆满完成1021个村委会、175个社区的换届选举工作。深入开展“五五”普法宣传教育。积极支援四川地震灾区，全市共捐款6843万元。监察、统计、审计、民族、宗教、外事、侨务、港澳、对台、人事、妇女儿童、红十字、人防、气象、地震、史志、档案、法律援助、殡改等工作均取得新进展。

——民生工作得到改善。超额完成了社会保险扩面任务，解决了困难企业职工的基本养老保险和医疗保险问题；征缴各项社会保险费12.47亿元，增长31.2%；支付各项社保待遇9.29亿元，增长28.65%；社保基金累计结余21亿元，增长26.3%；实现了社会保险基金市级统筹，启动了城镇居民基本医疗保险和被征地农民养老保险；参加农村合作医疗人数314.7万人，参合率达到98%；参加城镇居民基本医疗保险人数42.4万人，超额完成了省下达的任务。全面完成低保扩面工作，低保覆盖人口14.9万人，全年支出低保资金1亿多元，基本实现了应保尽保。统筹做好劳动力就业再就业工作，新增就业岗位7.4万个，城镇登记失业率为2.86%，零就业家庭继续保持动态为零。廉租住房保障资金得到落实，市区第一批廉租住房建设已启动。城乡居民生活水平稳步提高，城镇单位在岗职工人均工资25799元，增长9.5%；农民人均纯收入4887元，增长14.1%。平安清远建设深入开展，治安防控体系逐步完善；打击非法传销成果显著，摘掉了传销活动“重灾区”的帽子。积极预防和妥善处理各种社会矛盾，解决了一批历史遗留和疑难信访问题，实现了全国“两会”、奥运会、残奥会期间到京零上访。安全生产形势平稳好转，完成了省下达的安全生产控制指标任务；各类事故得到有效控制，连续八年未发生重特大火灾。大力开展打假治劣，加大重点产品质量和食品安全专项整治力度，确保了产品质量和食品安全。

各位代表，2008年，我们克服了年初严重雨雪冰冻灾害的侵袭，以及9月份以来历史罕见国际金融危机的冲击，在科学发展、和谐发展的道路上迈出了新的步伐。这些成绩的取得来之不易，是全市人民在市委正确领导下团结拼搏、扎实工作的结果，是市政府见事早、行动快、创造性贯彻落实中央宏观经济政策的结果，是市人大、市政协监督支持的结果，是港澳台同胞、海外侨胞和社会各界关心支持的结果，是兄弟地区尤其是对口帮扶地区热心帮助的结果。在此，我代表市人民政府，向全市人民、人大代表、政协委员，向各民主党派、工商联和无党派人士、人民团体，向驻清解放军、公安干警、武警和消防官兵、国家和省驻清单位，向海外侨胞、港澳台同胞以及所有参与、支持和关心清远建设的海内外朋友，表示衷心的感谢，并致以崇高的敬意！

在充分肯定成绩的同时，我们也清醒地看到，当前我市经济社会发展还存在不少困难和问题：受金融危机冲击，企业生产经营困难，外贸出口形势严峻，房地产市场收缩；确保经济增长、加快扶贫开发及加强民生保障财政支撑压力加大；产业结构不尽合理，企业自主创新能力不强，发展方式仍然比较粗放；生态环境约束加大，推动科学发展任务艰巨等等。我们必须高度重视，采取有效措施，切实加以解决。

2009年工作安排

2009年将是国际经济形势继续发生深刻变化的一年，发展面临的不稳定不确定因素增多。受全球经济明显放缓及我市粗放型发展方式累积的深层次矛盾和问题影响，我市经济发展面临前所未有的严峻挑战。但同时，也面临重大机遇。国家和省扩内需促发展各项政策措施的全面实施，特别是上升为国家级战略规划的《珠江三角洲地区改革发展规划纲要》的正式实施，将进一步加快我市融入珠三角步伐。加上我市新引进项目的逐步落实及重点项目的全面推进，投资对我市经济增长的拉动作用将进一步增强。经过20年的发展，我们已积累了一定的经济基础，工业化、城镇化正处于加速扩张阶段，基础设施建设、产业发展和居民消费拓展潜力较大。只要我们坚定信心，迎难而上，抢抓先机，就一定能变压力为动力，化危机为机遇，确保经济社会平稳较快发展。

2009年工作的总体要求：全面贯彻落实党的十七大、十七届三中全会和省委十届四次全会精神，按照市委五届七次全会的部署，以科学发展观统领经济社会发展全局，以“三促进一保持”（促进提高自主创新能力、促进传统产业转型升级、促进建设现代产业体系、保持经济平稳较快发展）为总要求，以实施《珠江三角洲地区改革发展规划纲要》为新动力，坚持在发展中调整，在落实中推进，千方百计加大投入，实现扩大内需保增长；千方百计调整结构，实现产业优化升级；千方百计推动新农村建设，实现扶贫开发新突破；千方百计优化发展环境，实现经济社会可持续发展；千方百计改善民生，实现社会和谐发展；千方百计提高工作效能，实现政府各项工作目标。

根据市委全会要求，今年全市经济社会发展的主要预期目标是：生产总值增长16%左右，人均生产总值增长13.8%；单位生产总值能耗下降3.6%，二氧化硫和化学需氧量削减量完成省定目标，全社会固定资产投资增长18%以上，外贸出口总额有所增长，社会消费品零售总额增长18%以上，居民消费价格总水平涨幅控制在3.8%以内，地方一般预算收入增长20%以上，城镇登记失业率控制在3.8%以下，人口自然增长率控制在7.8‰以内，在岗职工平均工资增长10%，农村居民人均纯收入增长8%。

具体抓好以下八项工作：

一、切实抓好扩大内需，增强经济发展动力

加快重大项目建设。抓好国家和省扩大内需各项措施的落实，着力抓好交通基础设施、民生、社会保障等项目的申报工作，争取更多项目纳入国家和省的计划“笼子”。积极推进城际轨道、广（州）乐（昌）、二（连浩特）广（州）及佛（山）清（远）从（化）高速清远段、清远抽水蓄能电站、侨远电厂扩建、500千伏库湾（清城）输变电等一批交通、能源基础设施建设，广清城际轨道力争9月底前动工。加快清远水利枢纽、飞来湖、大燕河拦河坝及乐排河截流蓄水工程建设，启动市区第二水厂建设。大力推进静福公园扩建、南山公园、连江西路等一批市政工程建设。切实抓好引进项目的落实，提高项目履约率和资金到位率。

千方百计促进消费。进一步增加城乡居民收入，提高消费能力。狠抓食品安全工作，着力改善消费环境，积极引导和扩大消费。加快推进中汽南方国际汽车城、清远义乌商贸城、农产品批发市场等专业市场建设，继续推进“万村千乡”市场工程，大力实施“家电下乡”工作，挖掘消费潜力。加快廉租房等保障性住房的开发建设，抓好国家、省和市各项刺激房地产消费政策的贯彻落实，促进住宅产业健康发展。落实好国家培育汽车消费需求的各项政策，扩大汽车消费市场。

二、切实抓好结构调整，推动产业优化升级

提高支柱产业发展水平。按照对接《珠江三角洲地区改革发展规划纲要》要求，加快制定产业结构调整与优化升级实施方案。围绕提升建材、有色金属、电子、生物制药等支柱产业发展水平，主动接受珠三角产业转移，进一步延长和完善产业链条，培育一批特色主导产业，打造一批名牌产品，扶持一批企业上市，推动产业结构的调整优化。加快产品质量检验检测平台建设，为支柱产业发展提供技术保障。

大力发展第三产业。进一步抓好旅游基础设施建设，大力推行旅游标准化。积极参与省旅游综合改革先行先试工作，带动第三产业发展。积极引进和培育大型流通企业集团，支持鼓励城市大中型零售企业和农村小型企业联合，促进农村商贸流通业发展。积极推进电子商务、会展、金融、保险等现代服务业发展，提升餐饮、旅业、娱乐等传统服务业层次。强化专项治理，进一步规范市场秩序，为第三产业的发展营造良好环境。

积极扶持中小企业发展。进一步加大财税政策扶持力度，减少行政事业规费，完善社会服务体系，优化中小企业发展环境。加强对中小企业经济运行的监测与分析，扶持发展中小企业融资和信用担保体系，帮助中小企业解决实际困难。引导和支持中小企业建立完善现代企业制度，提高竞争力。加大国家、省和市帮扶企业各项政策的宣传力度，增强企业家信心。

优化对外贸易结构。切实抓好国家、省和市各项外经贸政策的落实，重点支持一般贸易出口、品牌产品和优势特色产品出口，促进加工贸易转型升级，指导企业实现出口转内销。加大对20家重点农业企业的扶持力度，加快水果、蔬菜等农产品出口基地建设，提高农产品出口比重，培育新的外贸出口增长点。加紧开拓东盟、南美、非洲、俄罗斯等新兴市场。

三、切实抓好农村改革发展，促进农民持续增收

提高农业产业化水平。积极引进和培育产业关联度高、具有较强带动力的农业龙头企业，大力发展农民专业合作组织。稳定粮食生产，进一步加快名优家禽、蔬菜、水果等优势农产品基地建设，加快生态养殖小区建设，将清远建设成为珠三角的农产品生产供应基地。进一步加大农业科技研发、引进、推广和转化力度，提高农产品科技含量。继续抓好农业品牌的培育和打造，逐步完善农产品质量安全监测体系，大力推广标准化生产，积极开发无公害农产品和绿色食品。办好清远市首届“沙糖桔节”。大力发展农副产品加工业，拓展农产品流通网络，延长农业产业链，促进农业增效、农民增收。

加快新农村建设。将新农村建设与扶贫开发、乡村管理结合起来，落实配套资金，加快农村饮水、道路、沼气、电力等基础设施及文体设施、休闲场所建设，开展健康向上的文体活动，提高农民生活质量。加大农村环境卫生整治力度，逐步建立农村卫生保洁长效机制，进一步改变农村面貌。开展创建宜居村镇活动。

进一步加大扶贫开发力度。深入实施扶贫开发“规划到户、责任到人”，加强帮扶项目效果跟踪，确保扶贫开发取得实效。积极争取社会力量参与互助资金帮扶，帮助贫困村、贫困户解决生产发展启动资金问题。以中职院校为依托，继续推进智力扶贫，使每个贫困户掌握1至2门农业实用技术。继续抓好革命老区建设、农村安居工程、产业化扶贫、整村推进和面上各项扶贫工作。积极推进高寒山区、少数民族地区移民搬迁工作，促进农村人口向中心镇集中。

积极推进农村改革。进一步稳定和完善土地承包关系，创新土地流转模式，逐步建立农村土地流转交易平台和服务机构，按照依法自愿有偿原则，鼓励和支持利用土地流转进行规模开发，加快推进农村资源向规模经营集中。落实耕地保护行政问责和离任审计制度，稳定和完善土地承包关系。巩固农村税费改革成果，加大山林纠纷调处力度。积极稳妥推进林权制度改革。加快基层农业科技推广体系改革。深化农村信用社改革，积极推进农村金融体系建设。积极开展农村住房保险工作。

四、切实抓好“双转移”工作，推进区域协调发展

进一步提升园区建设水平。创新融资体制，放大省示范性产业转移园扶持资金效应，进一步完善佛山（清远）产业转移工业园基础设施及相关配套服务设施，发挥龙头示范带动作用。加快广东华侨经济产业园、广东民族工业园、佛山禅城（清新）产业转移工业园等重点园区建设，争取“台资企业产业转移园”落户我市。进一步完善园区产业规划，明确产业发展定位。围绕延伸产业链、推动上下游产业配套发展，加大优质企业引进力度，主动融入珠三角现代产业分工配套，加快形成特色鲜明、配套完善的产业集群，使我市成为珠三角先进制造业配套基地和新兴产业的成长地。加快园区环保基础设施建设，加强园区生态环境保护，严禁高污染企业进入园区。

加大招商引资力度。突出产业招商和园区招商，争取更多优质项目、品牌企业落户我市。切实做好现有企业的帮扶工作，促进增资扩产，以商引商。组织举办好第二届“佛山（清远）产业转移推介会”等招商引资活动，积极搭建招商引资平台。继续实施“走出去”战略，积极参与各种形式的经贸交流活动。争取今年对台招商有新突破。

加快劳动力培训与转移。大力推进各类就业培训载体建设，进一步加大劳动力培训力度，加快培养一批市场急需的中高级技工和熟练工人，着力提升本土人力资源竞争力。切实抓好转移就业公共服务平台建设，积极拓展劳务合作渠道，加大农村劳动力输出力度，确保完成年度劳动力转移任务。

五、切实抓好发展方式转变，夯实科学发展基础

增强企业自主创新能力。实施技术标准战略，支持企业参与国家标准、行业标准的制（修）定，鼓励企业采用国际标准或国外先进标准组织生产。大力推进产学研合作，支持有条件、有实力的企业组建技术中心或工程中心，争取今年申报认定1-2家省级技术中心或工程中心。推动重点领域、重大项目技术创新，力争在先进制造业、环保与资源综合利用等关键技术领域实现突破。支持和鼓励企业加大先进管理方式和技术力量的引进力度，用高新技术和先进适用技术提升传统产业。继续实施品牌带动战略，引导企业提升产品质量，争创品牌，以质取胜。

深入推进重点领域改革。继续深化行政审批制度改革，进一步减少和规范行政审批。积极稳妥推进事业单位分类改革。深入推进华侨农场改革，加快解决华侨农场历史遗留问题。完善国有资产运营和监管体系，推进国有资产保值增值。深化财政改革，推进基本公共服务均等化建设，进一步拓展国库集中支付改革范围，推行公务卡结算，试行财务核算信息集中监管改革；继续深化“收支两条线”管理改革，增强财政资金绩效评价工作，提高预算编制的科学化、精细化水平。进一步深化价格改革，建立并完善有利于市场经济发展的价格体制和机制。进一步加快停业整顿城市信用社退出市场工作。继续做好股权托管工作。

加强节能减排和环境保护工作。切实抓好重点企业节能降耗工作，支持现有高能耗企业加快技术改造，确保完成单位生产总值能耗年度目标。严格执行主要污染物排放总量控制制度，严格污染源在线监控，确保已建治理设施的企业稳定达标排放。积极主动配合省广业公司，加快各县（市、区）污水处理厂建设，确保年底前建成投入运行。加强重点区域、重点行业污染整治，巩固大燕河、乐排河流域污染整治成果。加快市区生活垃圾焚烧发电项目建设，实现生活垃圾处理无害化、减量化、资源化。继续深入开展环保专项行动，加大违法排污企业的清理和整治力度，严厉打击焚烧工业废物行为。加强医疗废物和危险化学品监管。建立长效机制，加快生态重点工程建设，深入实施“城乡清洁工程”。继续推行集约、节约用地。

六、切实抓好各项事业发展，提高公共服务水平

坚持优先发展教育。大力推进义务教育均衡发展，重点发展高中阶段教育，从今年起，实施普及高中阶段教育规划任务。加快推进中等职业技术教育结构调整，扩大中等职业教育规模。进一步推进与珠三角中职学校的联合办学，落实初中毕业生输送珠三角职校就读工作。继续开展教育创强工作，力争评估4-5个市级教育强镇，创建2-3个省级教育强镇，建设市一级学校8所，增加中小学优质学位8000个以上。进一步提高“普九”水平。加快市特殊教育学校建设。加强学前教育。启动高技能人才培养工程，继续推进清远职业技术学院的改革，促进内涵建设上新台阶。积极鼓励多种形式办学，引导民办教育健康发展。切实加强教师队伍建设，进一步提高教师待遇，积极稳妥解决好代课教师问题。

加快发展医疗卫生事业。积极推进医疗卫生体制改革，进一步优化医疗卫生资源配置，将更多医疗卫生资源向农村、贫困山区、少数民族地区倾斜。加大城乡基层医疗、卫生、计生服务体系建设，着力提高农村医疗卫生服务水平。进一步完善新型农村合作医疗制度，切实稳定参合率，全面提高保障水平。加快在建公共卫生基础设施建设，启动市中医院门诊急诊大楼建设，确保人民医院新院区建成并投入使用，进一步完善公共卫生体系和基本医疗服务体系。

积极推进文化、体育、计划生育等社会事业发展。深化文化体制改革，大力发展文化事业。加快启动市体校综合训练大楼、市群众艺术馆、市博物馆、市图书馆等一批文化体育基础工程，积极推进文化信息共享工程、流动图书馆、基层综合文化站建设，切实抓好第三批“农家书屋”示范点建设。进一步繁荣文艺创作，鼓励创造一批文艺精品力作。继续抓好文化遗产保护传承工作。积极开展“扫黄打非”工作。加快乡镇广播电视网络升级改造，进一步提高农村广播电视覆盖率。推进市区广播电视数字化工程建设。继续举办好全国或国际性的漂流、龙舟等体育赛事。办好第四届市运会，做好2010年省运会备战工作。认真贯彻实施新修订的《广东省人口与计划生育条例》，深入开展打击“两非”（非医学需要胎儿性别鉴定、非医学需要终止妊娠）行动，确保“两无”活动取得新成效。进一步加强信息化建设，关注网络民声，提高公众网上办事率。继续抓好“全国文明城市”创建活动，启动“全国卫生城市”创建工作。切实抓好全民国防教育，办好“民兵之家”，加强人民防空和民兵预备役建设，巩固“省双拥模范城”创建成果。

七、切实抓好民生工作，促进社会和谐稳定

进一步扩大就业再就业。加快完善面向城乡劳动者的人力资源市场体系、公共就业服务体系。落实积极的就业政策，加大社区和公益性就业岗位开发力度，多渠道扩大就业。完善鼓励自主创业的扶持政策和服务体系，以创业带动就业。进一步完善就业援助机制，重点帮助就业困难群体、大中专毕业生就业。深入实施农村青年、退役军人、农村低学历妇女、农民工等职业技能培训工程。加大劳动争议调处和劳动保障监察力度，维护劳动者合法权益。积极开展创建劳动关系和谐企业活动，促进劳动关系和谐稳定。

健全社会保障和救助体系。建立健全社会保险、社会救助、社会福利、慈善事业相衔接的城乡居民社保体系，进一步扩大社会保障覆盖面，提高保障水平。抓好以城乡低保为重点的社会救助工作，确保动态管理下的应保尽保。积极探索农村养老补贴制度，抓好被征地农民养老保险、优抚对象和城镇居民医疗保障工作。做好五保户、高龄贫困归难侨和残疾人等困难群体的救助工作。建立优抚对象抚恤优待补助标准自然增长机制。强化社会保险基金监管，确保基金安全和保值增值。积极做好养老保险城乡统筹试点工作。

切实抓好安全生产。严格落实安全生产责任，深入开展安全生产专项整治，坚决查处无证照生产经营行为，遏制各类重特大事故发生。加强安全生产宣传教育，提高全民安全意识。巩固产品质量和食品安全专项整治成果，建立健全食品安全预警应急机制，确保产品质量和食品安全。

全力维护社会稳定。以排查整治治安重点地区和突出治安问题为重点，进一步改善社会治安环境。保持打击传销高压态势。着力抓好平安县（市、区）和平安村（社区）创建活动，进一步加强社会面、乡镇（街道）和村（社区）治安防控体系建设。严厉打击生产销售假冒伪劣产品行为。完善社会矛盾纠纷排查调处机制，积极防范和妥善处置各种群体事件。进一步完善应急体系建设，解决群众反映强烈的突出问题，促进社会和谐。

八、切实抓好作风建设，提高政府行政能力

抓落实是今年政府工作的首要任务，也是完成全年目标的根本保证。各级政府要将主要精力和举措集中到抓落实上来，切实提高政府行政能力，并以此检验深入学习实践科学发展观活动的具体成效。

围绕强化责任抓落实。将保增长、扩内需、调结构、促民生各项责任细化，层层分解到领导成员和具体部门，明确完成时限和基本要求，确保落实到位。市政府常务会议要专题研究、督办重点工作、重大项目的落实，加强专项工作的协调推进。加强对责任落实情况的检查督促，并及时兑现奖惩措施。

围绕转变作风抓落实。继续减少和规范行政审批项目，简化办事程序，提高办事效率。严格控制会议、文件的数量、规格和规模，减少“文山会海”。厉行节约，努力降低行政开支，确保由政府举办的开放性晚会、展览、庆典、论坛活动比去年实现“四个减半”；严格控制一般性行政经费支出，确保公务购车和用车经费、会议经费、公务接待费用、出国（境）费用、办公经费预算实现“五个零增长”。继续加大机关作风整顿力度。

围绕提升服务抓落实。强化服务理念，充分发挥各行业商会、协会的作用，切实帮助企业解决实际困难。进一步加强电子政务建设，深入推行政务公开，及时公布政府规范性文件和政务动态，让市民更多了解与参与市政工程和民生工程建设，提高公共服务效能和质量。认真办理人大、政协的重要议案提案和信访热点问题。加强对宏观经济和社会发展形势的监测，及时研究制定应对措施。

围绕廉政建设抓落实。继续抓好“五个一流”（争创一流的领导班子、一流的干部队伍、一流的工作业绩、一流的干部作风、一流的干部形象）活动，推进政府机关和公务员队伍廉政建设，切实抓好党风廉政建设各项规定及“廉风和畅”活动的落实。着力抓好中央、省和市扩内需促发展政策措施执行情况的督促检查，强化政府投资项目和专项资金管理。严厉查处工程建设、土地管理、矿产资源开发、环境保护、金融和国企改革领域的违法违纪行为。

同志们，做好今年的工作任务艰巨，责任重大。让我们更加紧密团结在以胡锦涛同志为总书记的党中央周围，高举中国特色社会主义伟大旗帜，深入贯彻落实科学发展观，进一步解放思想，振奋精神，锐意进取，为推动清远经济社会在更高层次上实现又好又快发展，继续争当山区科学发展排头兵而努力奋斗！
